# Automated chemical synthesis. Part 4: Batch-type reactor automation and real-time software design

**DOI:** 10.1155/S1463924686000238

**Published:** 1986

**Authors:** Daniel F. Chodosh, Kenneth Kamholz, Sidney H. Levinson, Robert Rhinesmith

**Affiliations:** Smith Kline & French Laboratories 1500 Spring Garden Street Philadelphia Pennsylvania 19101 USA


Journal of Automatic Chemistry/*Journal of Clinical Laboratory Automation*, Vol. 8, No. 3 (July–September 1986), pp. 106–121

### Automated chemical synthesis. Part 4: Batch-type reactor automation and real-time software design

**Daniel F. Chodosh, Kenneth Kamholz, Sidney H. Levinson and Robert Rhinesmith**

*Smith Kline & French Laboratories, 1500 Spring Garden Street, Philadelphia, Pennsylvania 19101, USA*

The ability to efficiently and comprehensively evaluate synthetic routes at their earliest stage of development is especially important in pharmaceutical process research and development and chemical engineering laboratories [1 and 2]. The evaluation and optimization of typically complex synthetic sequences (involving many reaction parameters) frequently entails bench-scale experimentation of a highly repetitive, manpower-intensive nature. While systems for pilot and production scale batch-type reactor automation are widely [3–6] available most bench-scale laboratory automation research has been applied to continuous-flow reactor schema [7–10]. Often, the first opportunity to design and test automation software is at the pilot scale and, therefore, requires large quantities of developmental chemical materials for experimentation. These pilot scale studies, undertaken at more advanced stages of process development, have little opportunity to affect the prerequisite process discovery and process research effort. The application of automation technology at earlier stages of the research and development programme would provide greater opportunities to guide synthetic route selection and optimization. Further, the automation models developed at this early stage could be applied to the subsequent pilot and production scale laboratories. The authors' research focuses on the design and construction of a computer-controlled bench-scale batch-type chemical reactor capable of self-directing experimentation, reaction optimization and extensive data acquisition [11–16].

An automated batch-type reactor apparatus must be capable of delivering precise amounts of chemical reagents and solvents to a vessel, controlling the reaction time and temperature, and performing on-line chemical analyses of the reaction solution. Costly reagents must be conserved; thus a basic design concern is the minimization of the vessel size. The precision and accuracy of reagent/solvent delivery systems, temperature control systems and the chemical analysis system are, of course, of fundamental concern. Furthermore, to permit unattended operation fail-safe features involving hardware and software self-checking and error trapping must assure instrument integrity.

A functional block diagram of the automated chemical synthesis is presented in figure 1. Real-time control is provided by a Digital Equipment Corporation (Maynard, Massachusetts, USA) MINC LSI 11/2 computer system (see below). The MINC computer was selected in part, for its interface modularity and ease of access to these interfaces. The computer has been configured with 2 × 16 bit digital (i.e. TTL) output, 1 × 16 bit digital input, programmable clock, four-channel 12 bit D/A, four-channel 12 bit A/D and preamplifer (for resistance-to-voltage and current-to-voltage signal transduction) interfaces. The TTL signals available through the digital output interfaces, while capable of interfacing to semiconductor logic, cannot directly couple to devices such as motors or solenoids. A special optoisolator interface (figure 2) has been designed, which provides signal translation (TTL–→ 120 VAC; TTL–→ variable VDC) to permit facile coupling of non-TTL ON/OFF devices to the computer system. The optoisolator interface provides fail-safe default signals to the synthesis apparatus, in the event of failure of either the MINC computer hardware or the failure of a software driven watch-dog timer. A full description of this interface has been previously reported [17–18].

### Temperature control

The temperature control system balances the effects of resistive heating elements with the heat removed via an internal glass coil fitted to the reactor vessel through which coolant is circulated. The heating and cooling systems respond quickly to computer control signals and have sufficient capacities to effectively respond to thermal anomolies of chemical reactions (exotherms, endotherms). To minimize local heating effects, two resistance heating elements are employed: the exterior of the vessel is coated with a thermal element (ACE Glass, Inc., INSTATHERM, Vineland, New Jersey, USA) that provides even heating over a large surface area (*c.* 40 W over 440 $mm^2$) and an immersion heater fitted with a grooved stainless-steel sheath (Watlow, Inc., St. Louis, Minnesota, USA; 60 W over an effective surface area of 104 $mm^2$). A proportionating heater control circuit allows dynamic control of the heater duty cycle via a single D/A interface signal (figure 3). The cooling system is fully automated with control provided for the flow of coolant through the internal vessel coils and for the temperature of the cold bath reservoir (figure 4). These systems and the automation schema we employ have been previously described [19].

### Liquid trafficking system

To provide accurate and precise manipulation of liquid materials over a wide viscosity range, Hamilton Digital Diluters (Hamilton, Inc., Reno, Nevada, USA) are used;

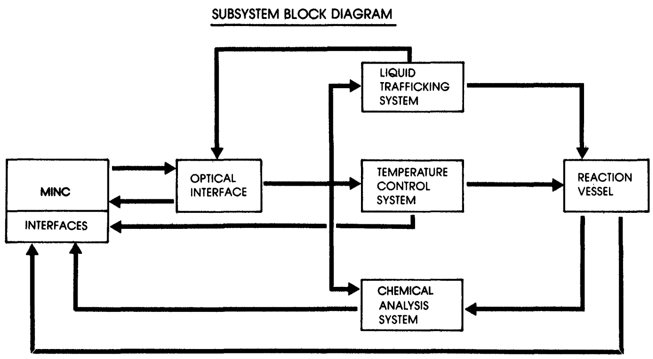

*Figure 1. Functional block diagram of synthesis system.*

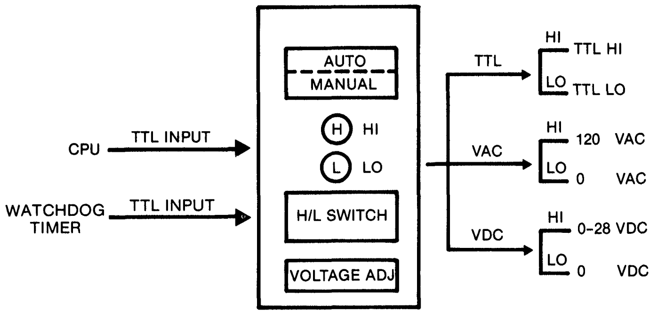

*Figure 2. Optoisolator interface.*

the logic boards of the units have been completely redesigned to allow direct control of syringe movement and valving via computer driven TTL signals. Each unit consists of two independent syringes mated to a common valve (figure 5), which simultaneously selects fill or inject positions for both syringes: SYRINGE (A)-Line (A1), SYRINGE (B)-Line (B1); SYRINGE (A)-Line (A2), SYRINGE (B)-Line (B2) respectively. In the automation design the function of this valve has been retained, and, in fact, the valve positioning signal is used as the direction select line for syringe movements (figure 6).

The movements of the syringes themselves are completely decoupled – each syringe is driven by a separate high torque stepper motor, which in turn is directly controlled by TTL signals from the computing system. In this manner the automation software can determine the maximum syringe velocity (a function of liquid viscosity and syringe size), so as to prevent errors in delivery due to back-pressure effects, and can correctly manipulate syringes of different capacities during dilution operations. These units are equipped with strain sensors that prevent damage to the unit when attempting to move past the mechanical top-of-travel limit. The logic board makes use of this strain switch signal to decouple the drive pulse-train at the mechanical limit; further, more complete advantage of this feature is taken during the initialization procedure. At initialization, the computer sends an exaggerated pulse-train to all syringes, attempting to send each syringe past the actual hardware limit. From their initialized, top-of-travel, positions the automation software then tracks the position of each syringe in the system. The position of the valve is computer selected by a TTL signal, while actual positioning is controlled by

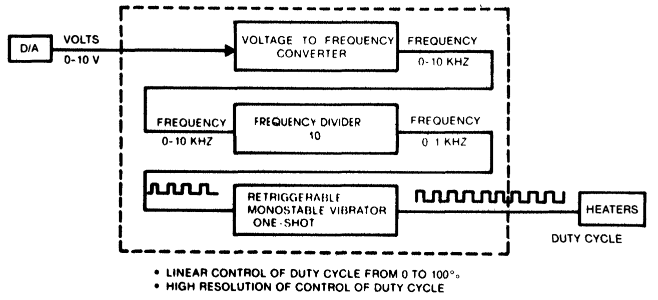

*Figure 3. Proportionating heater control circuit.*

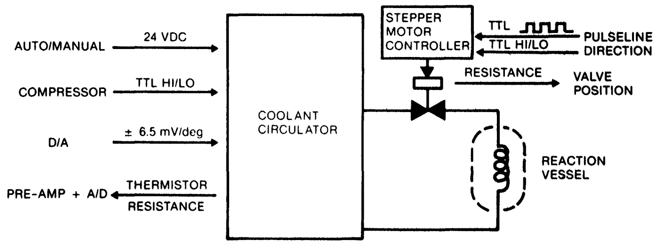

*Figure 4. Coolant control system.*

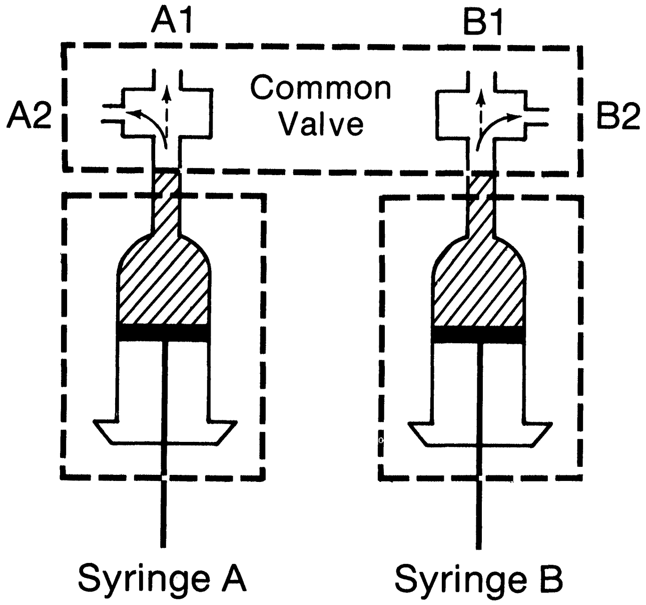

*Figure 5. Dual syringes with common valve.*

hardware on the logic board. The mechanical valve position is then reported out to the computer system via TTL signals. The automation software, therefore, provides for the generation of the valve position select signal, a suitable delay to permit settling of the valve to the new mechanical position and verification of the valve position via sense lines. The software further provides an error handling ability to attempt to 'unstick' a valve before aborting the experiment in progress. An electronic schematic of the syringe logic board is shown in figure 7.

The syringe systems have been tested over a wide range of liquid viscosities in order to determine the effects of delivery speed on accurcy and reproducibility (figure 8). Studies by Smith Kline & French correlate with the vendor's reports and demonstrate the ability to manipulate

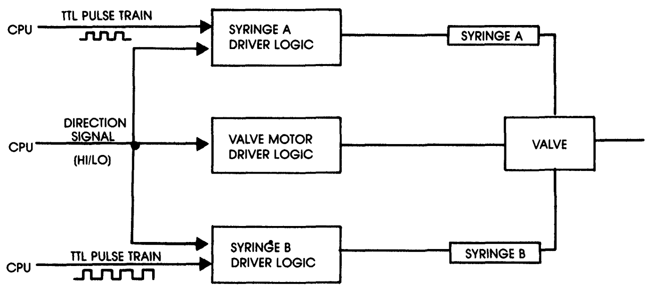

*Figure 6. Syringe control signals.*

samples with errors in delivery on the order of 0·5% mass delivered over the range of viscosities of interest. In the automation scheme four syringes are employed: (1) reagent/solvent delivery to reactor vessel; (2) drainage of reactor vessel between experiments and during washings; (3) reaction aliquot removal; and (4) diluent delivery for aliquot dilution preceding on-line chemical analysis. The capability to select and deliver reagent(s) and solvent(s) is provided with a single syringe through the use of a fully automated six-port rotary valve (Rheodyne, Berkeley, California, USA) interposed between the syringe and the reagent/solvent reservoirs (figure 9). The valve is pneumatically actuated, advancing one position each time a TTL position advance signal (operating a 12 V DC solenoid) is accepted from the computing system. The rotary valve is fitted with a magnetic armature (figure 10) and an annular ring of six Hall-effect magnetic sensors (Sprague Electric Co., Worchester, Massachusetts, USA). At position 1, a TTL sense signal is driven HIGH permitting the MINC computer to determine a reference position. A search for the reference position is executed in the start-up initialization and in error recovery procedures. During experimentation the automation software tracks the valve position from the reference position and the number of position advances pulses issued.

The initialization procedure assures that the lines between the radial rotary valve ports and their respective reagent/solvent reservoirs are filled and that the 'fill' line between the delivery syringe and the central valve port contains solvent. In operation, solvent is delivered to the vessel between each reagent delivery step such that reagents do not mix in the 'fill' line. The final material delivered to the reactor is always solvent so that the apparatus is ready for the washing procedure that follows each experiment. For a two reagent/one solvent experiment the delivery sequence entails Reagent A – 1/2 Solvent – Reagent B – 1/2 Solvent. Reagent reservoirs are maintained at reduced temperature as a precaution against decomposition.

The application of a second syringe is quite straightforward. The syringe 'fill' line connects at the bottom of the reaction vessel to the reactor drainage port; the syringe 'inject' line simply connects to the waste stream. A passive drain (for example gravity drain) proved unreliable during the early stages of our automation studies; this active drainage procedure allows more rapid emptying and washing between experimental runs thereby contributing to instrument throughput.

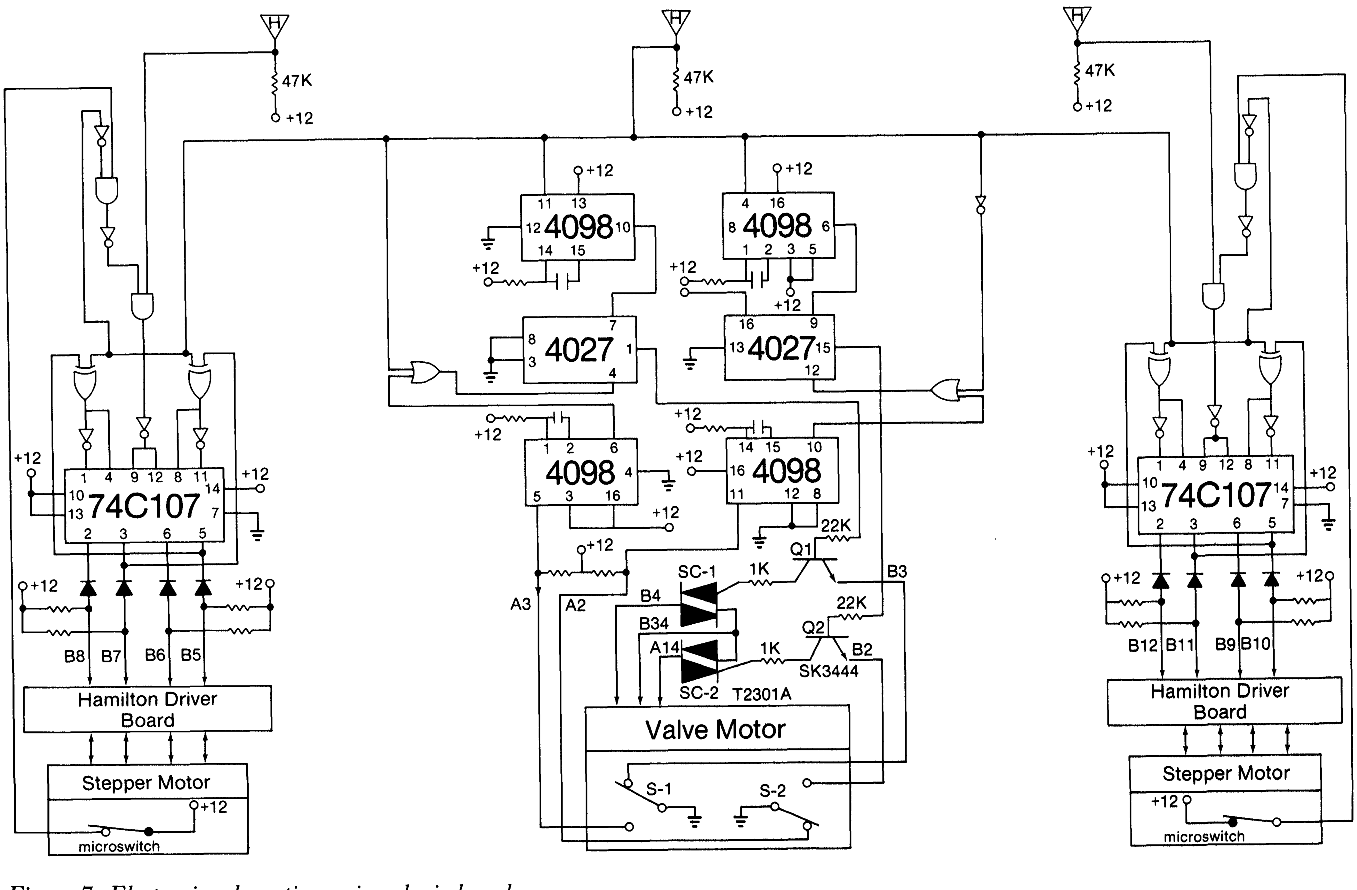

*Figure 7. Electronic schematic: syringe logic board.*

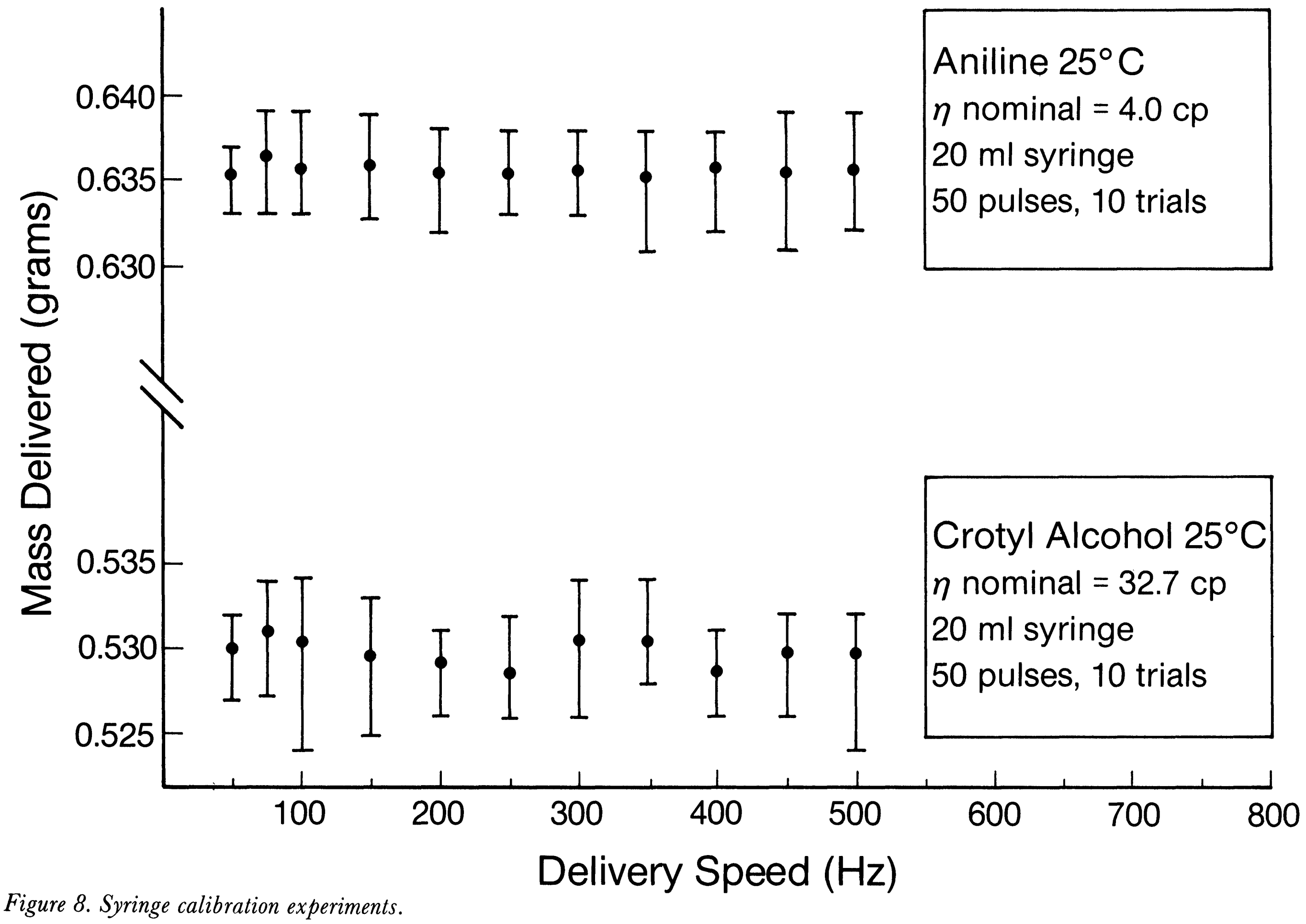

*Figure 8. Syringe calibration experiments.*

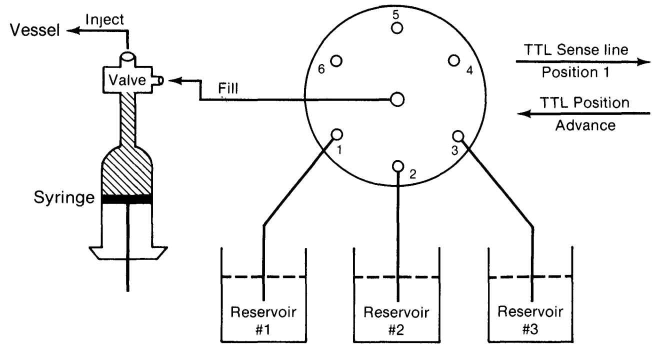

*Figure 9. Six-port rotary valve with syringe.*

Two additional automated six-port rotary valves are provided; these valves are used in tandem for intermediate storage of diluted reaction aliquots pending chemical analysis (figure 11). The time required to execute an on-line analysis of a reaction aliquot will depend upon the analytical method itself and the specific parameters of a given method for a specific chemical analysis. For example, UV/VIS or IR spectroscopic analysis, if appropriate, will provide extremely rapid analytical results; the method now employed, High Performance Liquid Chromatography (HPLC) requires significant time to execute, the analysis time being a function of numerous experimental parameters (column size, flow rate, diluent, chemical components of the reaction aliquot). The scheme permits the synthesis apparatus to sample, dilute and store reaction aliquots pending the availability of the HPLC system. If, for example, an experimental system requires a 15 min analysis time while it is necessary to sample once every 10 min (say, for a kinetic determination) the samples are stored in diluted form and passed to the analytical system when it becomes available (the diluent syringe, with appropriate six-port valve control, is used to move the sample to the injection loop).

In order to achieve a higher level of fail-safe integrity, optical flow cells are being designed to monitor the movement of materials through the liquid lines. These cells present TTL signals to the MINC computer (digital input interface) to allow absolute and direct verification of proper valve port selection and syringe movement. In this manner the automation software can detect reservoir depletion, malfunction of the valve or syringe units and can further assure the integrity of the lines themselves. Prototypes of these optical monitors have been tested in the Smith Kline & French laboratory.

### Chemical analysis

An on-line chemical analysis system has been integrated into the synthesis apparatus thereby 'closing the loop' and permitting automated execution of sequential and self-directed experiments [20–24]. HPLC has been selected as the method of choice, based upon its versatility

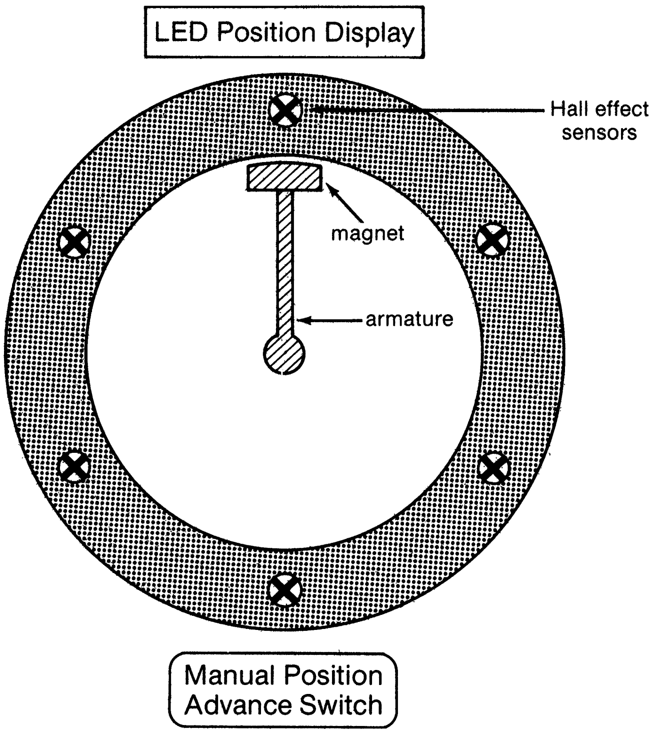

*Figure 10. Position sensing in rotary valve assembly.*

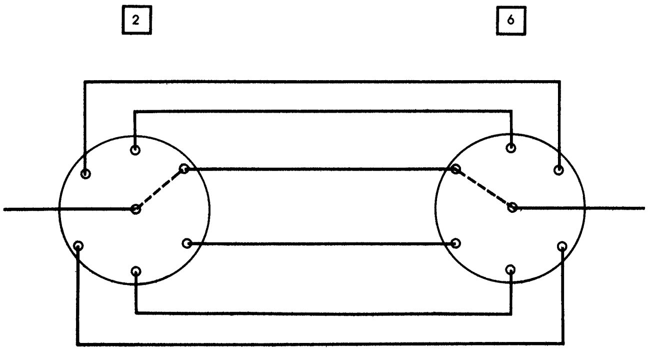

*Figure 11. Tandem six-port valve arrangement for aliquot storage.*

and, typically, the availability of certified analytical procedures concurrently developed in the course of chemical R&D programmes. As required, reaction aliquots are periodically removed from the vessel, diluted and introduced into the analysis system. A block diagram of this system is presented in figure 12. The sampling syringe removes a reaction aliquot of sufficient volume to clear the 'fill' line connecting it to the vessel. Under program control the diluent syringe is filled with the requisite volume of diluent for the dilution ratio (V : V) required. (This ratio is calculated from the reagent(s)/product(s) concentration and the predetermined column characteristics.) The syringe valve is switched to the 'inject' position; simultaneous movement of both syringes dilutes the sample while moving it to the analysis system. Both the speed of movement and the relative syringe capacities are factors in this dilution procedure; the automation software determines relative speed of movement for the syringes based upon their relative capacities and the dilution ratio desired. Typically the diluent syringe capacity is greater (by a factor of 10) than the sampling syringe capacity to facilitate the large dilution ratios often required for HPLC analysis. An algorithm has been developed that controls the simultaneous movement of these syringes at different speeds using the single programmable clock interface. Although the algorithm restricts the relative speeds to integer ratios (for example 1 : 1, 1 : 2, 1 : 3, . . .), the flexibility of the procedure is sufficient. A 'pulse buffer' is constructed consisting of alternating ON–OFF–ON–OFF– . . . data values. Starting with the first data value in the buffer the programmable clock places the next data value into the digital output register each time the required time period has expired (asynchronous data transfer). By 'masking' the ON values in this pulse buffer one syringe speed can be made an integer multiple of the other. For example, a pulse-stream directed to syringe A consisting of (ON–OFF–OFF–OFF–ON–OFF–OFF–OFF . . .) repeating values will provide an A : B movement ratio of 1 : 2. That is, for a fixed time period syringe B will receive twice as many TTL pulses as does syringe A; syringe B, therefore, moves at twice the speed of syringe A.

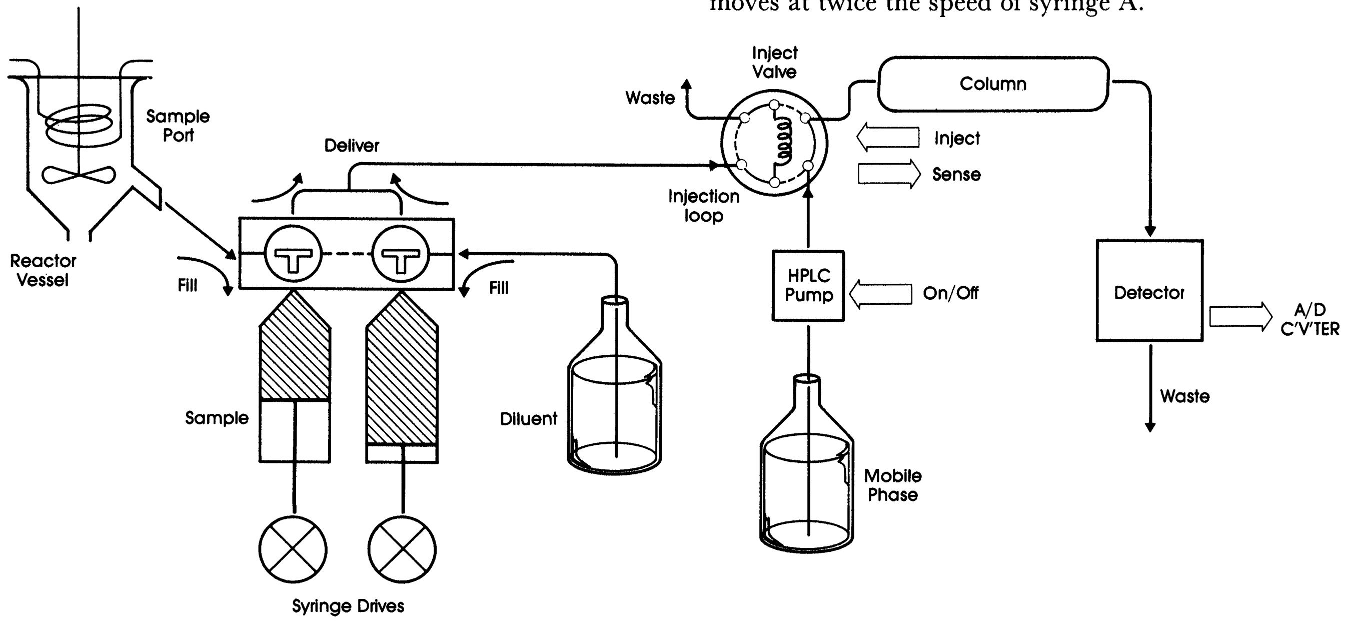

*Figure 12. Functional block diagram of on-line HPLC analysis system.*

The aliquot removal and dilution procedure is repeated several times to rinse the injection loop, all lines and the syringes; this also ensures that fresh diluted sample is presented to the analysis system. Once the HPLC injection loop is filled the diluted sample is injected onto the chromatography column by actuating a 12 V solenoid which in turn pneumatically drives the sample injector (Rheodyne, #7010A). The solenoid operation is initiated by a TTL signal from the MINC; the injection timing is fixed and is controlled by an on-board 'one shot' timing circuit. In this manner uniform sample injections are assured. A TTL sense line reports the injection operation to the computer to further assure proper operation allowing error detection and subsequent error recovery procedure, if needed, under computer control. Analytical data is obtained via an analogue input signal from the detector (Kratos/Schoeffel, GM770 Monochromator, SF770 Spectroflow Monitor, Westwood, New Jersey, USA) interfaced to the MINC through the preamplifier and A/D interface modules (TV/AU). Data acquisition rate is 10 samples/s. The detector provides an independent 0–10 mV output signal which is sent in parallel to a stand-alone recorder/integrator (Hewlett-Packard 3380A) for conventional recording and later comparison with MINC chromatographic analyses.

### Reactor apparatus

The batch-type reactor unit consists of a two piece, all glass assembly (head and vessel) of 75 ml approximate capacity. Liquid reagents/solvents are introduced through an inlet on the reactor head which feeds the inlet stream to a position proximal to the agitator propeller. To assure reliable agitation during experimentation, a mechanically driven agitator is used (figure 13). The MINC system controls the agitation using ON/OFF signals (120 VAC) while the speed is manually preset on the agitator motor drive. The reactor head is fitted with a thermistor probe (Thermometrics, Edison, New Jersey, USA) to provide temperature measurements to the MINC over the wide range of temperatures of interest. (The synthesis system is designed for use in the temperature range −20 °C to +150 °C.) Temperature measurement entails (1) the reading of the thermistor resistance; (2) conversion through the programmable preamplifer interface to a voltage signal; (3) conversion of the voltage to a digital value (A/D interface); and (4) calculation of the reactor temperature using the equation of the specific thermistor. The reactor head accommodates the immersion heating element and a reflux condenser used in elevated temperature studies. An inlet and an outlet for the vessel cooling coils are located on the reactor head assembly. The vessel exterior is coated with a thermal element and accommodates outlets for drainage and rection sample removal.

### Software design

The automation system hardware has been described above. The automation software is written in FORTRAN with real-time I/O performed by calls to the REAL MNC-11 software library. The operating system, Real-Time 11 Single User Foreground/Background, assigns higher execution priorities to time critical program sections and supports the full complement of input/output interfaces required for interfacing to the synthesis apparatus. The user memory, memory not taken up by I/O addresses or the operating system, is limited to *c.* 48k bytes. This available memory is not sufficient to wholly contain the complex multistep automation control

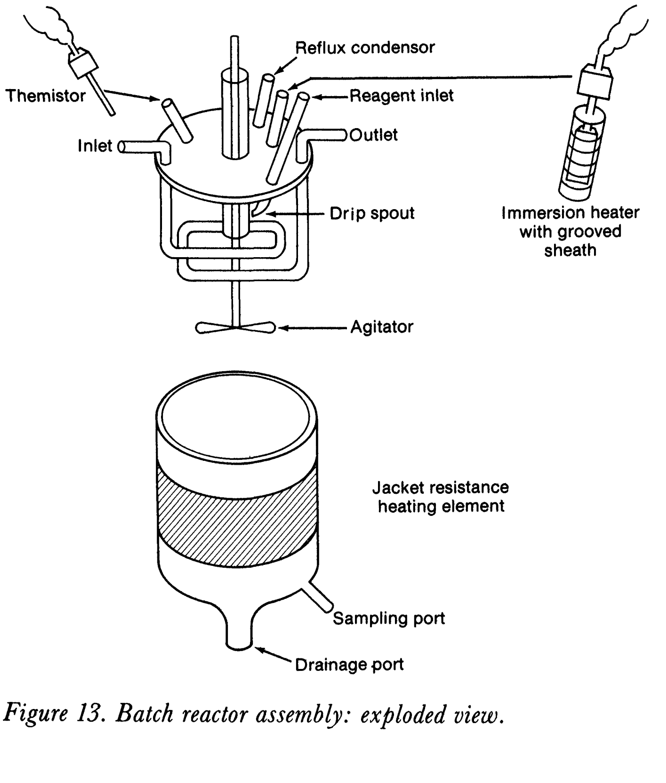

*Figure 13. Batch reactor assembly: exploded view.*

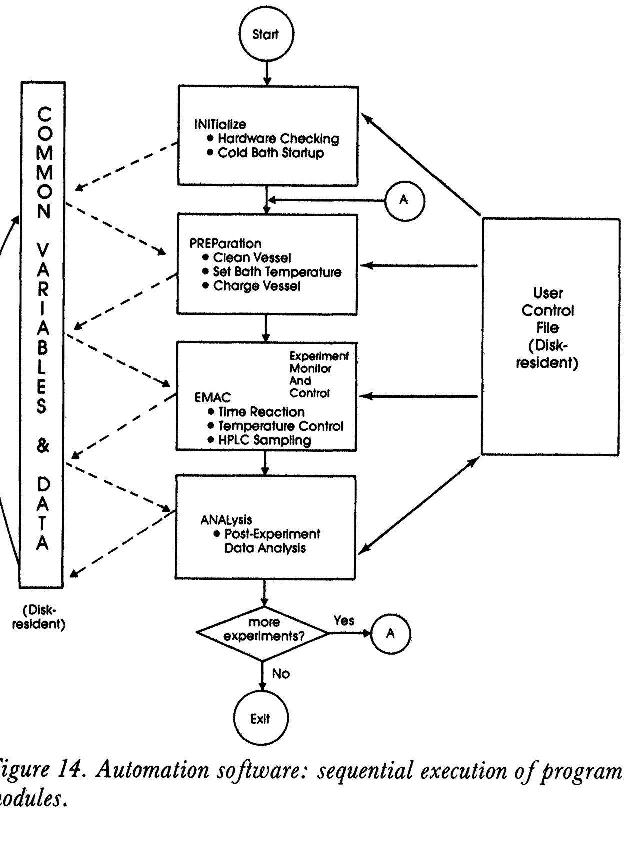

*Figure 14. Automation software: sequential execution of program modules.*

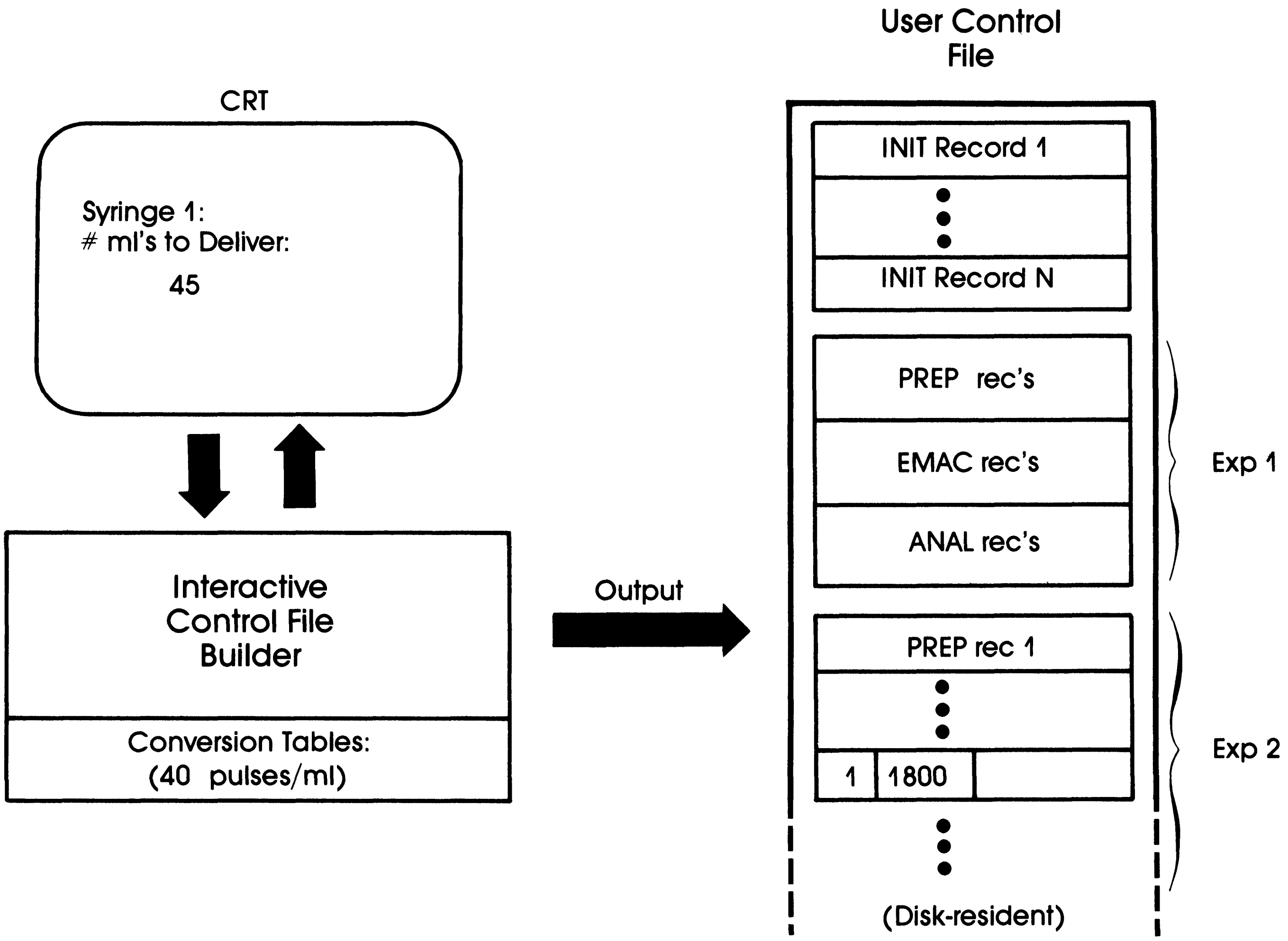

*Figure 15. Automation software: building the USER CONTROL FILE.*

software; independent program modules have therefore been designed, which, when used sequentially, execute a complete experiment. The execution sequence of the four program modules in this scheme is shown in figure 14. Since the modules execute independently (effectively, chaining under indirect file control) data variable values are not preserved or directly passed between modules. This scheme requires that the operating system supports indirect control files that enumerate the sequence of programs to be executed, and that all data be maintained on the permanent disk storage device is available to each program module as required. Each module when called into execution first reads COMMON data variables from the disk into memory and stores the latest values back to the disk immediately prior to terminating execution. Independent modules can be designed, tested and inserted into the scheme without modification to pre-existing software,

Experimental operating parameters are established in a disk resident USER-CONTROL FILE (cf. figures 14 and 15) and are available to all program modules. The user assembles information in this control file through the use of a text editor or via dialogue with an interactive program prior to beginning reactor operation. All subsequent experimental operations proceed without dialogue. Program modules access information, as required, from the USER-CONTROL FILE to direct their activities. In the file experiments are specified sequentially; a specific format is used to define a block of data variables which enumerate washing instructions, reagent stoichiometry, reaction temperature, sampling rate and the number of samples to be taken for a specific experiment. The next experiment is sequentially defined in the file with the identical format. Series of experiments can then be configured in the USER-CONTROL FILE for sequential, unattended execution. The synthesis instrument terminates all activity and shuts down operation in an orderly fashion when an EOF (end-of-file) is read from the file. This manner of operation is appropriate for factorial design experiments in which the user is able to delineate all experiments in the series. For a self-directed optimization (for example Simplex) new experiments are added to the control file by the ANALysis program module based upon the evaluation of preceding experimental determinations. Reaction operation continues by executing this newly defined experiment; this cycling continues until a termination condition is met (limited number of experiments; convergence of response to a preset tolerance).

Given the complex function of each program module it is not surprising that one would meet with great difficulty in designing FORTRAN programs to fit the available computer memory. Furthermore, complex functions can usually be divided into a progression of simpler

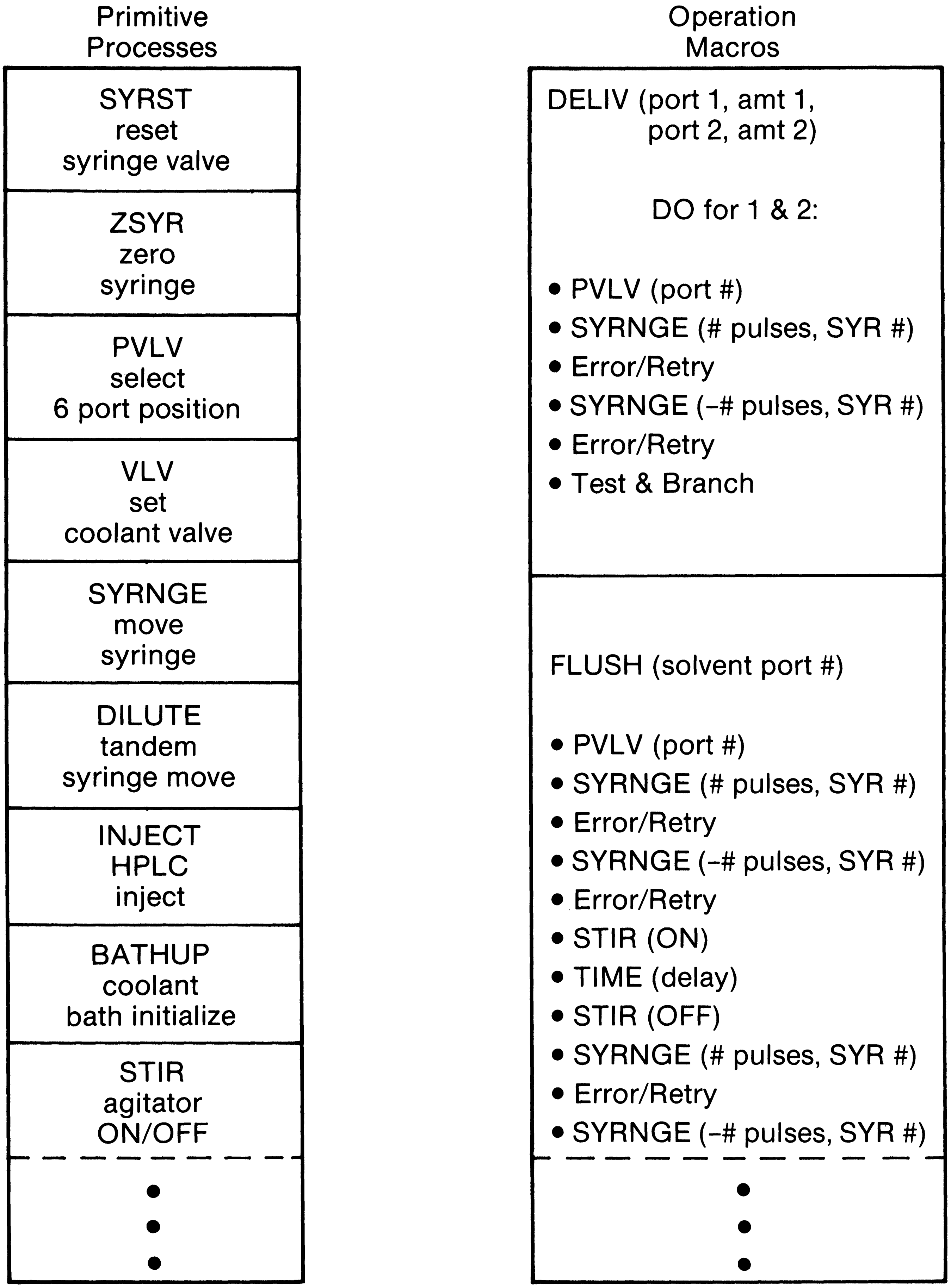

*Figure 16. Automation software: MACRO and PRIMITIVE libraries.*

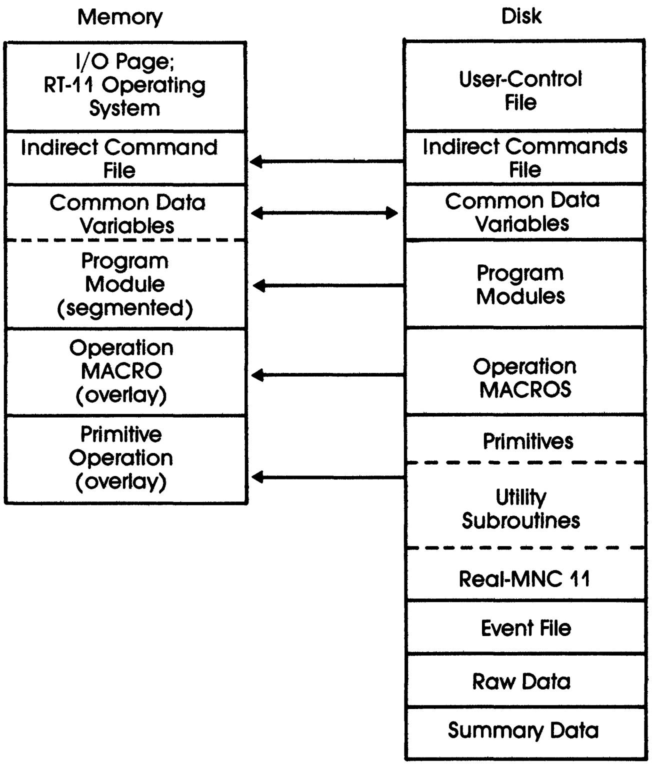

*Figure 17. Automation software: computer memory organization.*

operations. One can quickly ascertain that assortments of complex functions can be accomplished by recollection of simpler operations. Accordingly, a group of basic operations has been defined: 'primitive processes'. These PRIMITIVES have been collected into a disk resident library (figure 16). A PRIMITIVE operation addresses a specific hardware interface – the syringe drive board, six-port valve controller, temperature control board, etc. Examples of PRIMITIVES include: select a six-port valve position, move a syringe, select syringe valve position. When PRIMITIVES can be collected to perform a more complex task, this collection is saved as an OPERATION MACRO. These OPERATION MACROS are again collected into a disk library. For example, the DELIVERY macro calls the PRIMITIVES six-port valve select, move syringe (fill), and move syringe (inject). Any program module that requires the delivery of material calls the DELIVERY macro with the appropriate data arguments thereby avoiding redundant programming and conserving computer memory. More complex macros involve extensive looping, testing data variable and conditional branching to PRIMITIVES rather than a simple sequence of calls to primitives. To invoke this scheme we use an overlay technique which allocates regions of computer memory for the PROGRAM MODULES, OPERATION MACROS and PRIMITIVES. The organization of computer memory is shown in figure 17. The operating system (RT11) and the I/O page (device registers) are always memory resident leaving *c.* 48 kbyte for user applications. The indirect command file is read from the disk into memory by the operating system and directs the system to locate the first named PROGRAM MODULE (INIT) on the disk device, load it into the designated area of memory reserved for PROGRAM MODULES and begin execution. When execution is completed, the next named PROGRAM MODULE is read into that same memory are replacing the previous module. Whenever a new copy of a module is required the operating system must return to the disk device to fetch a copy and load it into the computer memory. In a similar fashion, a certain area of memory is reserved for OPERATION MACROS. When a PROGRAM MODULE calls for a macro, the disk library of operation macros is searched and a copy of the named macro is loaded into the specified memory area destroying any previously read macro. When the macro begins execution it in turn overlays PRIMITIVES, as needed, from the disk resident PRIMITIVES library into the memory area reserved for PRIMITIVES and executes the indicated PRIMITIVE. Throughout this procedure copies of the PRIMITIVES, OPERATION MACROS and PROGRAM MODULES remain undisturbed on the disk device. An 'EVENT' file is maintained on the disk device.

PRIMITIVES sequentially record their activities and all exceptional conditions (i.e. errors) by making entries to this EVENT file. The file is printed at the conclusion of each experimental procedure. PRIMITIVES return error codes to their calling OPERATION MACRO where error processing and correction (if possible) operations can be invoked. The libraries of statistical and scientific subroutines and the REAL MNC11 library, all provided by the vendor, are maintained on the disc (Utility Subroutines, figure 17) and include routines such as peak processing, file management and real-time I/O functions. These routines are overlayed into the PRIMITIVES memory area when invoked for execution by an OPERATION MACRO or a PROGRAM MODULE [25].

The INITialize and PREParation PROGRAM MODULES perform their automation functions through a series of sequential calls to MACROS (and therefore, to PRIMITIVES). The INIT module starts up the cold bath, the syringes are zeroed to their top-of-travel positions and the six-port valves set to their reference positions. PREP sets the coolant bath temperature, assures that the liquid transfer lines from the reservoirs to the radial ports of the six-port valve are filled, then cleans the vessel by delivering solvent to the reactor, agitating and draining the vessel. The vessel is then charged with the requisite stoichiometry of reagent(s)/solvent(s) as directed by the data values obtained from the USER CONTROL FILE.

The Experiment Monitor And Control (EMAC) MODULE is far less straightforward involving substantially more complex operations and control of the autosynthesis apparatus. Throughout the remainder of the experiment EMAC establishes and maintains the temperature control of the vessel and its contents; samples, dilutes and injects reaction aliquots on to the analysis system as required; and collects/interprets the chemical analysis data. A functionalized flowchart of EMAC is presented (figure 18) showing the PROGRAM MODULE itself and its associated interrupt processing and completion routines. As described above, the

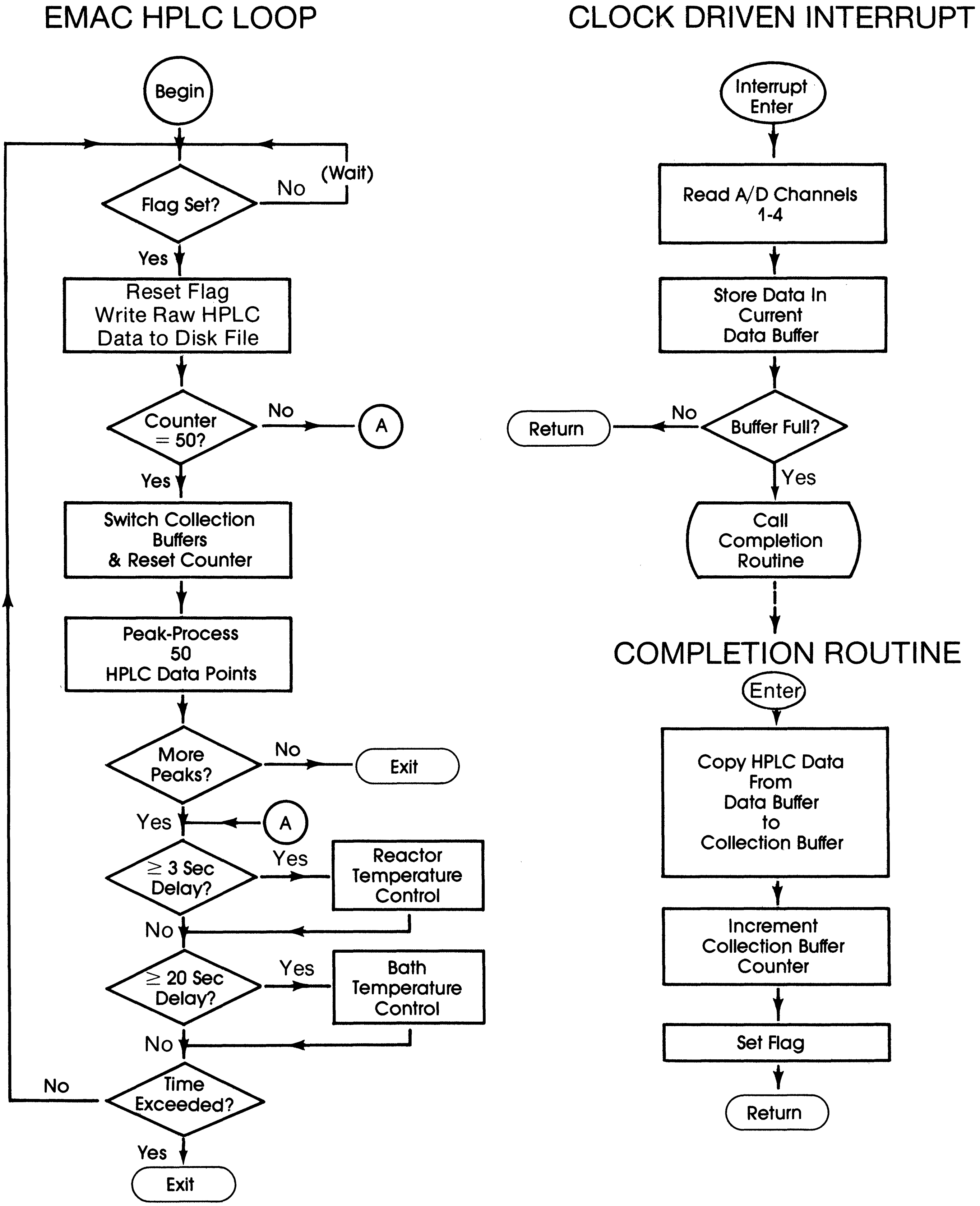

*Figure 18. Automation software: EMAC program module flowchart.*

sampling rate (10 Hz) is established by loading the programmable clock with the appropriate data value. Each time the requisite time period expires the clock generates an 'interrupt' causing the operating system to suspend processing of the current PROGRAM MODULE and to jump into the execution of the interrupt routine. When the processing of the instructions in the interrupt routine is completed execution of the PROGRAM MODULE

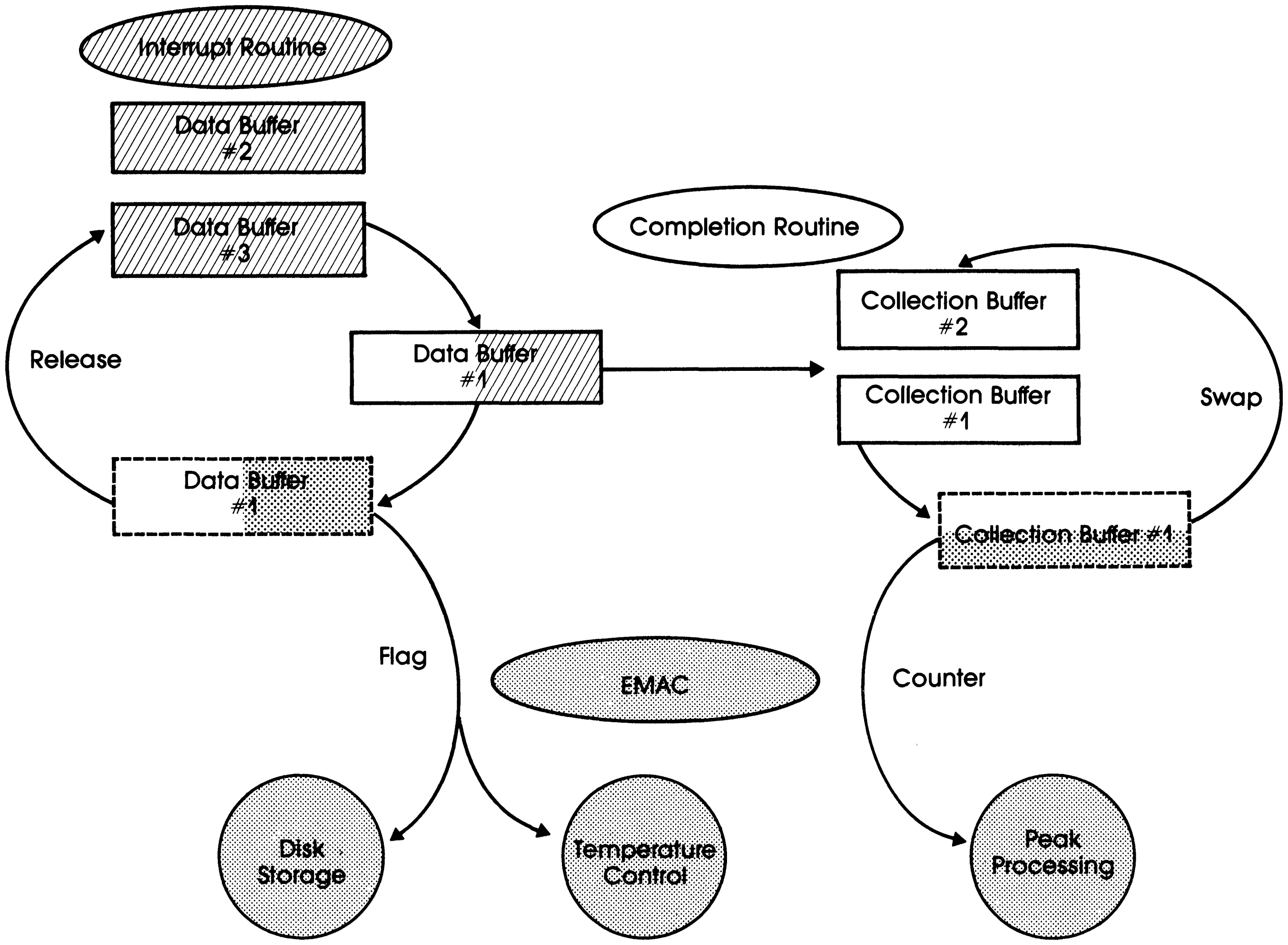

*Figure 19. Automation software: EMAC program module data buffer rotation scheme.*

resumes with the program statement that was suspended. Data values are therefore collected at the specified rate independently of the activities programmed into the PROGRAM MODULE itself. Each time an interrupt is generated the interrupt routine executes a 'sweep' of all four A/D channels, acquires the data values and stores these values in a data buffer. The A/D channels provide data from the HPLC detector, reactor vessel thermistor and the coolant bath reservoir thermistor; a fourth channel is available for pH analogue input. The interrupt routine stores the A/D data in a buffer of fixed size (variable data array, 4 × 10 words). When the data buffer is full (i.e. 10 interrupts, 1 s elapsed time) execution passes to a 'completion routine' that sets a flag that the PROGRAM MODULE can test to ascertain when a full data buffer is available for archiving. Three 4 × 10 word data buffers are available to the interrupt routine; these buffers are rotated so that a data buffer is available to the interrupt routine at all times while the PROGRAM MODULE manipulates the information in a filled buffer. These buffer rotations are illustrated figure 19. At position A empty data buffers are queued and are available to receive A/D values in the interrupt routine. Information has been accumulated into Data Buffer #1; after 4 × 10 values were obtained the completion routine was called to copy only the HPLC data from the newly filled data buffer to a collection buffer (50 values; position B in figure 19). The completion routine maintains a counter of the number of data buffers unloaded into the collection buffer an sets a flag indicating successsful operation. The interrupt routine is now accumulating A/D data into Data Buffer #2. The EMAC program module uses the completion routine flag and counter to direct its activities. The HPLC data is extracted from Data Buffer #1 and archived to a disk file when the success flag indicates the data buffer has been filled (position C). Temperature measurements are extracted from the data buffer and passed to temperature control algorithms which monitor and control the coolant bath reservoir temperature at 20 s intervals and the reactor vessel temperature at 3 s intervals. The data buffer is then released and requeued to receive new A/D data. This scheme assures that a data buffer is always available to the interrupt routine for data acquisition. The counter is tested to determine whether or not a full 50 value collection buffer is available for HPLC peak analysis (position D). When Collection Buffer #1 is filled the counter is reset to zero and the completion routine begins filling Collection Buffer #2. After Collection Buffer #1 has been filled it is numerically manipulated (digital filtering) and passed to a subroutine to identify peaks in the chromatogram. This latter function is non-time critical and can take place as long as there is always sufficient collection buffer space to unload the data buffers which participate in the time critical operation (interrupt processing). When the manipulation of the data contents of Collection Buffer #1 is completed it can be swapped with Collection Buffer #2 to continue this cycle. This rotation of buffers guarantees the availability of collection buffer space for the completion routine.

The operation continues until the appropriate termination condition is met: either the specified number of peaks have been collected from the chromatogram or the specified analysis time has elapsed. The ANALysis PROGRAM MODULE is then executed to perform (optionally) any indicated post-processing computations. Execution cycles to the PREP program module for the next experiment if indicated in the USER CONTROL FILE.

### Experimental designs

When one attempts to optimize a given step in a synthesis it is often useful to obtain a stability profile of the reaction mixture components. This profile can be constructed through a series of experiments which quantitate the component stabilities as a step function of the various reaction parameters: temperature, concentration and other reaction parameters often including but not limited to, pH, solvent composition and catalyst. This type of experimentation is inherently repetitious and by employing the hardware and software previously described can be fully automated.

Decomposition rate determinations were performed on the cephalosporin reaction components I and II.

(TSA = Tetrazole Sulfonic Acid)

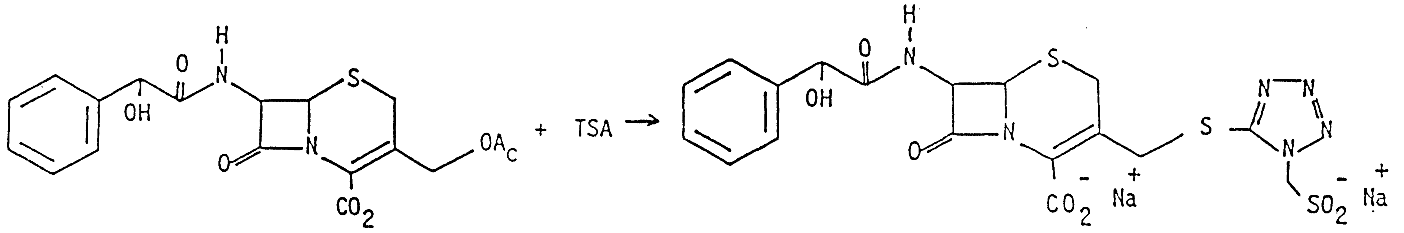

I II

An independently certified HPLC method [26] was employed to monitor these decomposition reactions. Preceding the cephalosporin studies; the reproducibility of the HPLC analysis system was established using a resorcinol (Baker Chemical Co., Phillipsburgh, New Jersey, USA) test system. A standard resorcinol solution was introduced into the reaction vessel and repeated chromatograms were obtained. The retention times for nine injections ($RT_{mean}$ = 3·85 min) varied by less that ±0·01 min (±0·3%). The integrated area ($counts_{mean}$ = 69 000) showed a standard deviation of 0·33% with a maximum absolute count fluxation of ±1% from the mean. The small error terms associated with this analytical method permits experimental design studies in which small changes in reaction parameters result in small but chemically significant changes in the chemical composition of the reaction mixture. Forty experimental determinations were performed entirely under program control. The procedure followed was:

(1) The reaction vessel was first cleaned by flushing with solvent and then emptied.

(2) Starting material was introduced to the vessel. A solution of standard concentration of each starting material was prepared, maintained at reduced temperature and available to the automated system via the six port valve assembly. For those experiments which required varying starting material concentrations, the appropriate volume of solvent was delivered via a third port to affect the dilution ratio required to attain the requisite concentration of starting material. For the pH dependent studies two reservoirs of starting material buffered at different pHs were available to the system and were mixed in the appropriate proportion to yield a starting material solution of the required pH. (Solution pH measurements were confirmed by manual checks.)

(3) The stirred reaction mixture was brought rapidly to the required temperature. Temperature control during the course of experimentation was typically ±0·1° of the setpoint.

(4) An initial aliquot was withdrawn and submitted for on-line chemical analysis.

(5) After succeeeding fixed time intervals, selected to permit several aliquots to be taken over the first half-life of the starting material, aliquot removal and chemical analysis steps were repeated.

(6) The experiment was terminated when either (*a*) a pre-established time period had elapsed, or (*b*) the chromatogram analysis indicated that the peaks of major interest had decreased in intensity below a pre-established percentage of their original value.

(7) The EVENT file was listed on the printer along with summary reports of peak retention times and peak areas for all analyses performed. These data files, as well as the raw HPLC data, were retained on the disc for future report writing and, if necessary, further off-line numerical analysis.

(8) If the USER CONTROL FILE indicated another experiment was to be performed, the entire sequence of steps was repeated. In this manner a series of experiments could be executed without user intervention.

In these initial studies the analysis of the decay rates were performed off-line; therefore the ANALysis program module was effectively bypassed though it still served to update cycle counters for subsequent passes through the USER CONTROL FILE.

Decomposition rates were evaluated with repect to initial concentration, temperature and pH (table 1). Generally,

### Experimental Design

| Exp. # | T°C | pH | Buffer | [ ] | # Trials |
|---|---|---|---|---|---|
| 1 | 35.0 | 5.0 | 0.15 N Acetate | 0.02 N II | 1 |
| 2 | 40.0 | ↓ | ↓ | ↓ | 2 |
| 3 | 45.0 | ↓ | ↓ | ↓ | 2 |
| 4 | 50.0 | ↓ | ↓ | ↓ | 2 |
| 5 | 55.0 | ↓ | ↓ | ↓ | 2 |
| 6 | 60.0 | ↓ | ↓ | ↓ | 2 |
| 7 | 60.0 | 5.0 | 0.20 N Acetate | 0.010 N II | 2 |
| 8 | ↓ | ↓ | ↓ | 0.025 N II | 2 |
| 9 | ↓ | ↓ | ↓ | 0.030 N II | 2 |
| 10 | ↓ | ↓ | ↓ | 0.075 N II | 4 |
| 11 | ↓ | ↓ | ↓ | 0.120 N II | 2 |
| 12 | ↓ | ↓ | ↓ | 0.150 N II | 2 |
| 13 | 60.0 | 4.8 | 0.15 N Phosphate | 0.02 N II | 1 |
| 14 | ↓ | 5.2 | ↓ | ↓ | 1 |
| 15 | ↓ | 6.8 | ↓ | ↓ | 1 |
| 16 | ↓ | 6.9 | ↓ | ↓ | 2 |
| 17 | ↓ | 7.6 | ↓ | ↓ | 1 |
| 18 | ↓ | 8.2 | ↓ | ↓ | 1 |
| 19 | 35.0 | 5.0 | 0.15 N Acetate | 0.04 N I | 2 |
| 20 | 45.0 | ↓ | ↓ | ↓ | 3 |
| 21 | 55.0 | ↓ | ↓ | ↓ | 3 |
| 22 | 65.0 | ↓ | ↓ | ↓ | 2 |

*Table 1. Experimental design.*

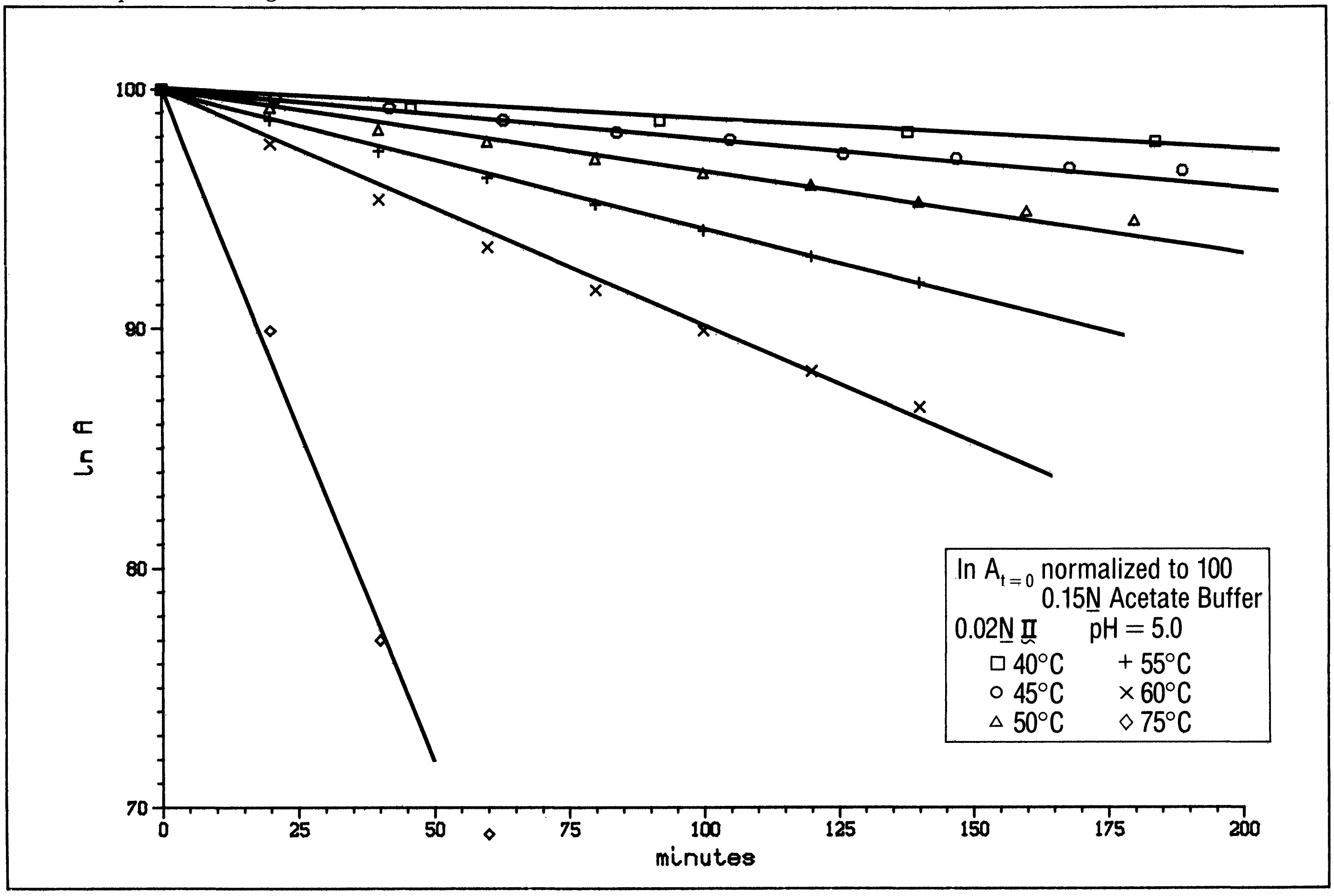

*Figure 20. LN(A) versus time II.*

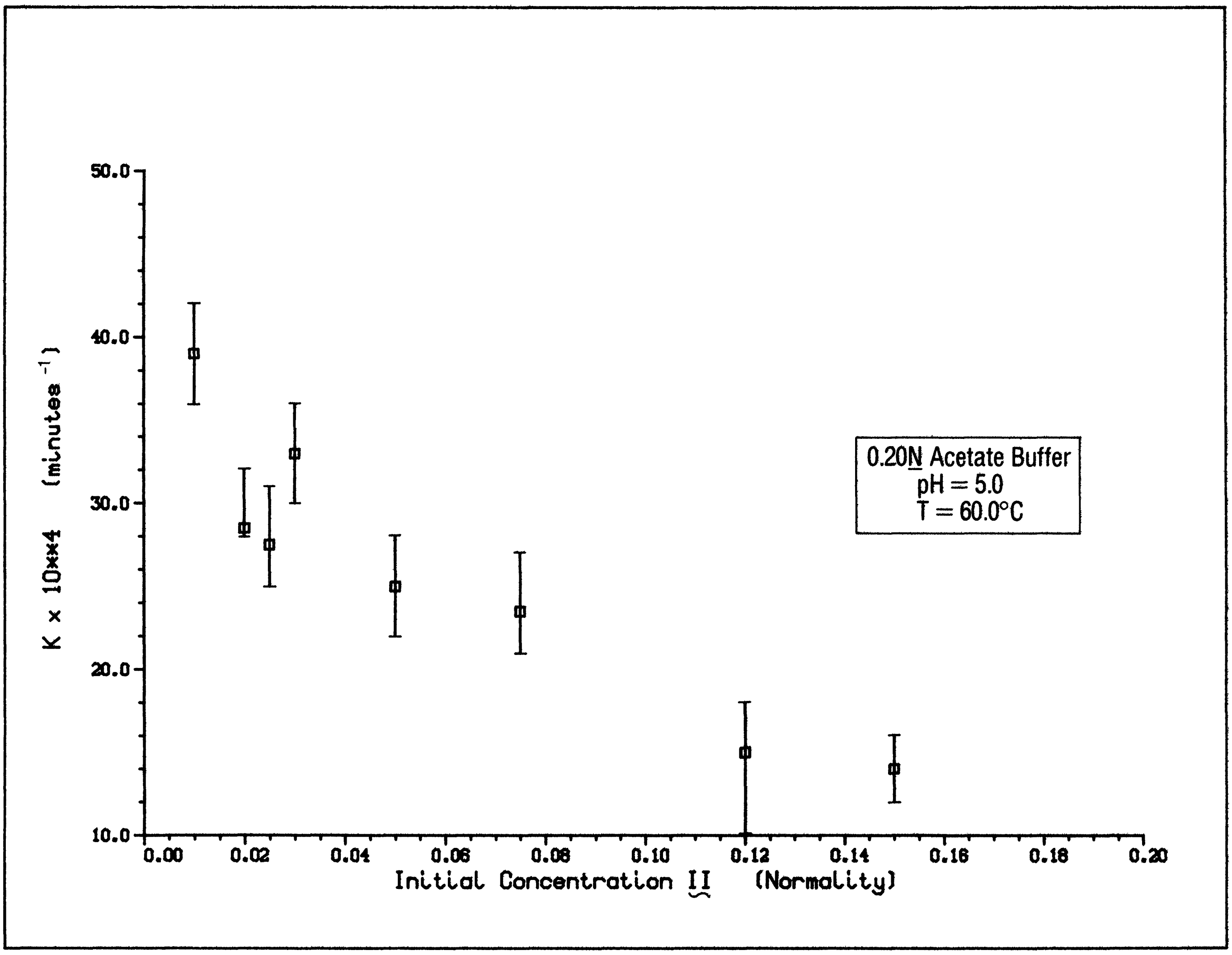

*Figure 21. Initial rate studies, II.*

the experimental design was selected to overlap previously performed non-automated experiments in order to compare the results of our automated technique with conventional methods. The analysis of the data is straightforward and was carried out with FORTRAN programs that utilized the data files from the automated experimental runs. The decomposition rates of both I and II approximately follow first order kinetics with pseudo first order rate constant (k, $s^{-1}$) obtained from least-squares fits to the equation,

$\ln(A) = \ln(A_o) - kt$ where $A_o$ is the peak area at time zero.

Plots of ln (A) versus time for elevated temperature studies (where it is convenient to obtain HPLC data over a time domain covering two or more component halflives) are shown in figure 20 and for I and II, respectively. The rate constant, k, varies from $5{\cdot}6 \times 10^{-4}\,min^{-1}$ (35°) to $13{\cdot}6 \times 10^{-3}\,min^{-1}$ (65°) for I (cf. Figure 20). For II, the rate constant varied from $3{\cdot}6 \times 10^{-4}\,min^{-1}$ (40°) to $14{\cdot}3 \times 10^{-3}$ (75°) (cf. figure 21). The results of the initial concentration studies of II are shown in figure 21. These results demonstrate a minor concentration dependence of the decomposition rate (*c* three-fold rate decrease over a 100-fold concentration increase) and indicate the shortcomings of our pseudo-first order model in which the role of the solvent is not described.

Plots of Ln K versus 1/T for I and II are presented in figure 22. Least-squares fits of the data for both I and II to the Arrhenius equation,

$k = A\,e^{-Ea/RT}$ A = pre-exponential factor
Ea = activation energy
RT = gas constant yield straight lines within experimental error, with

$A_I = 2{\cdot}2 \times 10^{12}\,min^{-1}$, $Ea_I = 22{\cdot}1$ kcal/mole;

$A_{II} = 4{\cdot}8 \times 10^{12}\,min^{-1}$, $Ea_{II} = 23{\cdot}1$ kcal/mole.

The pre-exponential factor for I, however, is significantly lower than that for II. A dissociative decomposition pathway, considered to be a solvolysis, is consistent with the equality of $Ea_I$ with $Ea_{II}$ (collision energy transfer from solvent) with lowering of the pre-exponential factor for II attributable to the steric hindrance of the substituent at C3.

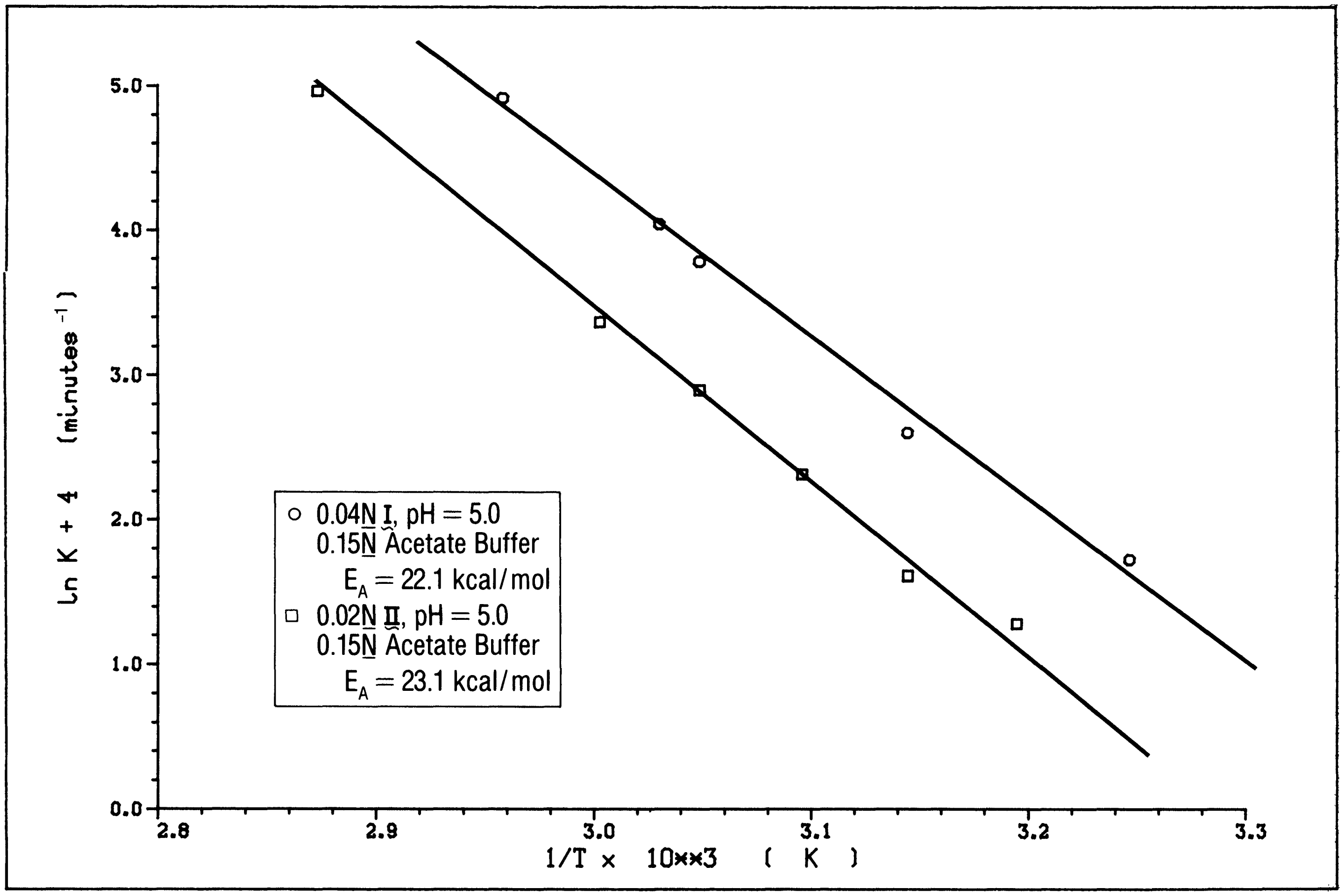

*Figure 22. Ln(K) versus 1/T I and II.*

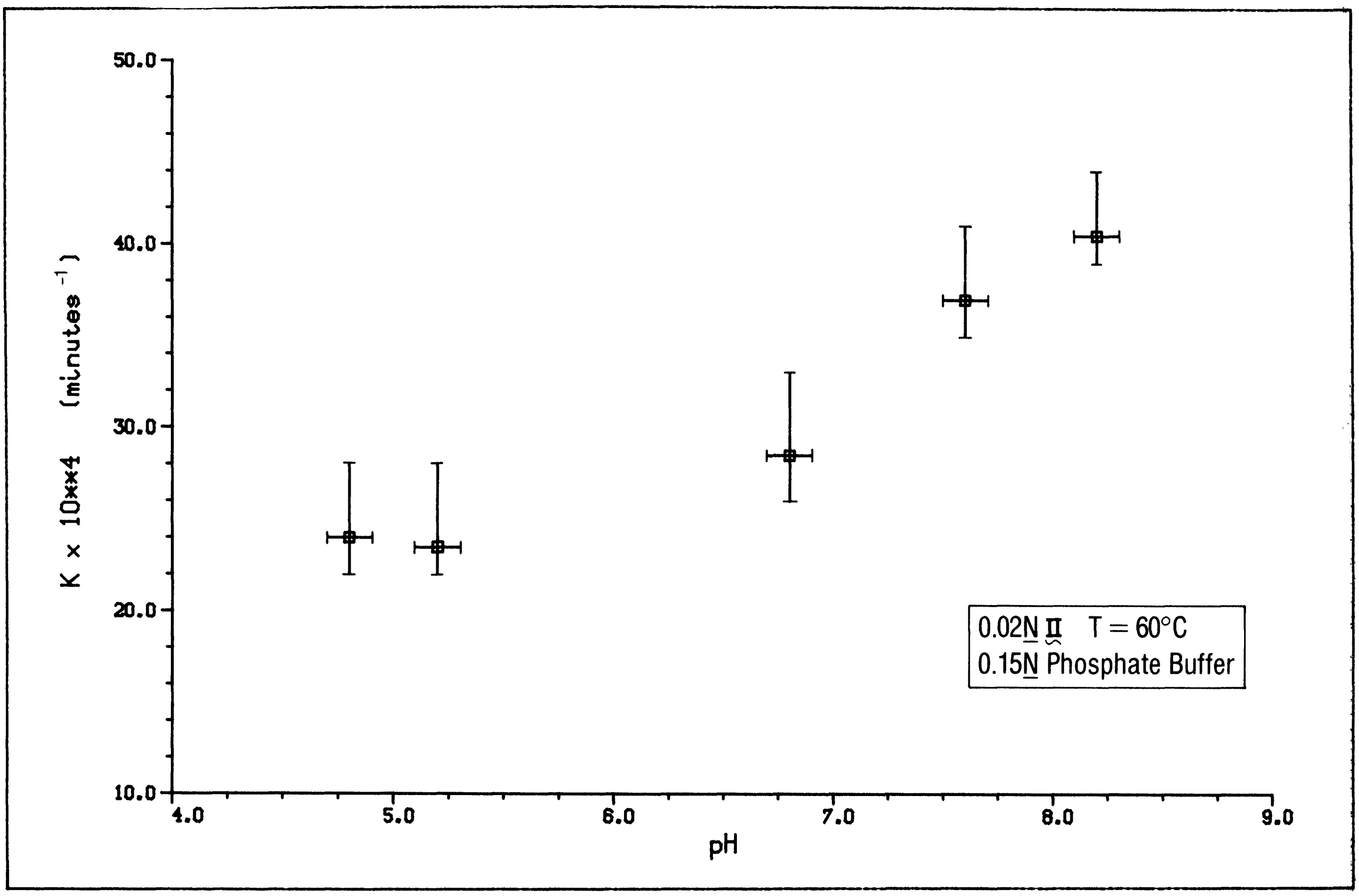

*Figure 23. II pH-dependent studies.*

The results of the pH dependent studies are presented in figure 23 and indicate the relative insensitivity of the decomposition kinetics to pH values in the range studied. The rate varies from $23 \times 10^{-1}$ min$^{-1}$ to $38 \times 10^{-4}$ min$^{-1}$ in the pH range 4·8 to 8·2.

### Discussion

This research effort focuses on laboratory scale batch-type automation research and fully complements and extends the automation studies of continuous flow reaction designs reported for both laboratory [9 and 10] and pilot [27] scale systems. As new microsensors and special microchips [28] become available the design and implementation timeframe for all such automated reactor schema will be significantly reduced thereby encouraging such experimentation. Interestingly enough, these research approaches require the participation of an interdisciplinary team, bringing together individuals with expertise in the chemical, chemical engineering and computer sciences. The building of such teams, in and by itself, has significant implications for research organizations where the team can and should participate in the global development of chemical processes from the earliest inception at the laboratory research level through to the production process.

### References

1. Walser, P. E. and Bertels, H. A., *American Laboratory,* (1982), 113.

2. Frazer, J. W., *Accounts of Chemical Research,* **7** (1974), 141.

3. Brodmann, M. T. and Smith, C. L., *Chemical Engineering,* **83** (1976), 191.

4. Kennedy, J. P., *Chemical Engineering Progress,* **77** (1981), 33.

5. Merritt, R., *Inst. Cont. Sys.* (1981), 34.

6. Garton, R. D., *Chemical Engineering Progress,* **77** (1981), 44.

7. Nagy, G., Feher, Z. and Pungor, E., *Analytica Chimica Acta,* **52** (1970), 47.

8. Spellman, R. A. and Quinn, J. D., AIChE Workshop in Industrial Process Control, Tampa, Florida (November 1974).

9. Frazer, J. W., Rigdon, L. P., Brand, H. R. and Pomernacki, C. L., *Analytical Chemistry,* **51** (1979), 1739.

10. Nakumara, R., Sakamoto, K., Sato, K., Niiyama, H. and Echigoya, E., *International Chemical Engineering,* **24** (1984), 536.

11. Winicov, H., Schainbaum, J., Buckley, Jr., J. T., Longino, G., Hill, J. and Berkoff, C. E., *Analytical Chimica Acta,* **103** (1978), 469.

12. Chodosh, D. F., Winicov, H., Buckley, Jr., J. T., and Berkoff, C. E., Computers at the bench, Belgian Pharmaceutical Soceity International Conference on Computers in Pharmaceutical Research, Namur, Belgium (November 1979).

13. Chodosh, D. F., 1978 Fall D.E.C.U.S. Symposium, Workshop of Laboratory Automation, San Francisco (November 1978).

14. Chodosh, D. F., Buckley, Jr, J. T., Levinson, S. H., Wdzieckowski, F. E., Weber, J. L., Winicov, H. and Berkoff, C. E., Automated synthesis: design of the chemical reactor hardware, 1981 Fall D.E.C.U.S. Symposium, Los Angeles (December 1981).

15. Dean, W. K., Heald, K. J. and Deming, S. N., *Science,* **189** (1975), 805.

16. Box, G. E. P., *Biometrics,* **10** (1964), 16.

17. Chodosh, D. F., Wdzieckowski, F. E., Schainbaum, J. and Berkoff, C. E., *Journal of Automatic Chemistry,* **5** (1983), 99.

18. Chodosh, D. F., Buckley, Jr., J. T., Longino, G., Schainbaum, J., Wdzieckowski, F. E., Winicov, H. and Berkoff, C. E., MINC interfacing, 1981 Fall D.E.C.U.S. Symposium, Los Angeles (December 1981).

19. Chodosh, D. F., Levinson, S. H., Weber, J. L., Kamholz, K. and Berkoff, C. E., *Journal of Automatic Chemistry,* **5** (1983), 103.

20. Frazer, J. W., Kray, A. M., Selig, W. and Lim, R., *Analytical Chemistry,* **47** (1975), 869.

21. Watson, M. W. and Carr, P. W., *Analytical Chemistry,* **51** (1979), 1835.

22. Deming, S. N. and Morgan, S. L., *Analytical Chemistry,* **45** (1973), 278A.

23. Hendrix, C., *Chemical Technology* (1980), 448.

24. Deming, S. N., *American Laboratory* (1981), 42.

25. The CHAINing instruction allows a program to call another program into execution while preserving a portion of memory to allow variable data values to be transferred between the two programs. This feature is available only for programs executing in the background mode in RT11.

26. Filan, J. and Dougherty, J., private communication.

27. Legrand, M., Roussel-UCLAF, Romaineville, France, private communication.

28. See: *Analytical Chemistry* (1985), **57**, 1298A.

